# Molecular mechanism of palmitic acid and its derivatives in tumor progression

**DOI:** 10.3389/fonc.2023.1224125

**Published:** 2023-08-09

**Authors:** Xitan Wang, Chaonan Zhang, Na Bao

**Affiliations:** ^1^ Hospital of Weifang Medical University, School of Clinical Medicine, Weifang Medical University, Weifang, China; ^2^ Weifang Medical University, Weifang, Shandong, China; ^3^ Jining First People’s Hospital, Jining, Shandong, China

**Keywords:** palmitic acid, cancer, molecular mechanism, apoptosis, P53

## Abstract

Palmitic acid (PA) is a saturated fatty acid commonly found in coconut oil and palm oil. It serves as an energy source for the body and plays a role in the structure and function of cell membranes. Beyond its industrial applications, PA has gained attention for its potential therapeutic properties. Modern pharmacological studies have demonstrated that PA exhibits anti-inflammatory, antioxidant, and immune-enhancing effects. In recent years, PA has emerged as a promising anti-tumor agent with demonstrated efficacy against various malignancies including gastric cancer, liver cancer, cervical cancer, breast cancer, and colorectal cancer. Its anti-tumor effects encompass inducing apoptosis in tumor cells, inhibiting tumor cell proliferation, suppressing metastasis and invasion, enhancing sensitivity to chemotherapy, and improving immune function. The main anticancer mechanism of palmitic acid (PA) involves the induction of cell apoptosis through the mitochondrial pathway, facilitated by the promotion of intracellular reactive oxygen species (ROS) generation. PA also exhibits interference with the cancer cell cycle, leading to cell cycle arrest predominantly in the G1 phase. Moreover, PA induces programmed cell autophagy death, inhibits cell migration, invasion, and angiogenesis, and synergistically enhances the efficacy of chemotherapy drugs while reducing adverse reactions. PA acts on various intracellular and extracellular targets, modulating tumor cell signaling pathways, including the phosphatidylinositol 3-kinase (PI3K)/protein kinase B (Akt), endoplasmic reticulum (ER), B Cell Lymphoma-2 (Bcl-2), P53, and other signaling pathways. Furthermore, derivatives of PA play a significant regulatory role in tumor resistance processes. This paper provides a comprehensive review of recent studies investigating the anti-tumor effects of PA. It summarizes the underlying mechanisms through which PA exerts its anti-tumor effects, aiming to inspire new perspectives for the treatment of malignant tumors in clinical settings and the development of novel anti-cancer drugs.

## 1 Introduction

In recent years, the global incidence and mortality rates of cancer have been on the rise, making it a major cause of death worldwide (1). With approximately 19.3 million new cases and around 10 million deaths annually, cancer poses a significant public health challenge (2, 3). It accounts for one in eight deaths globally, highlighting the urgent need to explore the pathogenesis of cancer and develop safe and effective anti-tumor therapies.

Palmitic acid (PA) is a common fatty acid that constitutes 20-30% of the total fatty acids in the human body (4).It can be obtained through dietary intake (5), or synthesized in the body through fat metabolism (6). Palmitic acid exhibits unique functional properties compared to other saturated fatty acids (7, 8). Research has shown that PA can induce endoplasmic reticulum (ER) stress, which is attributed to the increased saturation level of phospholipids within the ER (9).The saturation level of phospholipids in the endoplasmic reticulum affects membrane chemical and physical properties, including fluidity and protein-membrane interactions. These changes impact the activation of specific signaling pathways, such as the activation of endoplasmic reticulum-related sensors like protein kinase RNA-like endoplasmic reticulum kinase (PERK), inositol-requiring enzyme 1 (IRE1), and activating transcription factor 6 (ATF6) (10–13). Furthermore, palmitic acid can be transferred to target proteins by a group of enzymes known as palmitoyl transferases (PATs). These enzymes facilitate the attachment of palmitic acid to specific cysteine residues within target proteins. Palmitoyl groups form unstable thioester bonds with cysteine residues, allowing for reversible palmitoylation, which dynamically regulates protein function (14, 15). Palmitic acid plays a vital role in post-translational modifications, specifically protein palmitoylation, which influences protein localization, stability, and interactions, thereby impacting various cell signaling pathways and processes (16–18).

PA exhibits a broad range of pharmacological activities, including antiviral, anti-inflammatory, analgesic, and regulation of lipid metabolism (19, 20). Regarding its anticancer properties, PA has been shown to induce cell cycle arrest and promote apoptosis in human neuroblastoma and breast cancer cells (21, 22). Extensive research has been conducted to elucidate the anti-tumor mechanisms of PA, both in *in vivo* and *in vitro* studies. These mechanisms include inhibiting the proliferation and differentiation of tumor cells, inducing apoptosis, and suppressing tumor cell invasion and migration.

Despite these findings, a comprehensive summary of PA’s anti-tumor potential and its molecular targets in various cancers is lacking. Therefore, this paper aims to systematically review the literature on the anti-tumor effects of PA in recent years, focusing on the mechanisms and targets involved. The objective is to provide new insights and a theoretical foundation for the further development and application of PA in cancer treatment.

## 2 Inhibition of tumor cell proliferation in response to PA

Cell proliferation is a critical process in cell growth and differentiation (23).Abnormal cell proliferation is a key characteristic of cancer (24), making the inhibition of tumor cell proliferation a crucial strategy in cancer therapy (25).Notably, both *in vitro* and *in vivo* studies have demonstrated the potential of PA in effectively inhibiting the proliferation of various cancer cell types, including prostate cancer, gastric cancer, and breast cancer cells.

### 2.1 Cell cycle blockade

Tumor-associated cell cycle defects are often driven by dysregulation of cyclins and cyclin-dependent kinases (CDKs) (26, 27). CDK inhibitors (CDKis) such as p27 play a crucial role in inhibiting the activity of CDK-cyclin complexes (28). In the context of prostate cancer, Zhu et al. (29) discovered that PA could dose-dependently inhibit the growth of PC3 and DU145 prostate cancer cells. Further investigations revealed that PA significantly increased the expression of p27 while decreasing the expression of pRb and cyclin D1 in these cells, leading to cell cycle arrest at the G1 phase. These findings indicate that PA can inhibit the proliferation of prostate cancer cell lines by disrupting the cell cycle progression. Similarly, in a study by Hsiao et al. (21), PA was found to induce cell cycle arrest at the G2/M phase in human neuroblastoma cells through protein palmitylation, demonstrating its anti-proliferative effect on tumor cells.

### 2.2 Janus kinase-signal transducer and activator of transcription pathway

JAK-STAT pathway plays a crucial role in various cellular processes, including tumor cell recognition and tumor-driven immune evasion (30). Consequently, inhibitors targeting the JAK/STAT3 pathway have the potential to impede tumor cell growth and promote antitumor immune responses. STAT3 is a pro-inflammatory and oncogenic transcription factor associated with tumorigenesis, inflammation, and immunosuppression (31). In gastric cancer cells, STAT3 is typically activated and localized in the nucleus, exhibiting high expression levels (32). In a study by Yu et al. (33), immunofluorescence analysis revealed that PA could reduce the expressions of p-STAT3, p-JAK2, n-cadherin, and vimentin in gastric cancer cells. Furthermore, PA inhibited the nuclear localization of p-STAT3, indicating its potential to inhibit the proliferation and metastasis of gastric cancer cells by suppressing the JAK2/STAT3 immune signaling pathway. These findings suggest that PA exerts its anti-tumor effects in gastric cancer by targeting the JAK2/STAT3 pathway. By downregulating the activation and nuclear translocation of STAT3, PA may inhibit tumor cell proliferation, metastasis, and promote antitumor immune responses.

### 2.3 The phosphoinositide 3-kinase/protein kinase B pathway

PI3K/AKT signaling pathway plays a critical role in tumor development and metastasis (34, 35). In a study by Zhu et al. (29), it was observed that palmitic acid (PA) could inhibit the expression of PI3K-P110α and PI3K-P110β, as well as the phosphorylation of downstream effectors including PDK1, Akt, mTOR, p70S6K, and GSK-3β in a dose-dependent manner. This inhibition resulted in the blockade of prostate cancer cell proliferation. Huang et al. (36) discovered that PA reduces glucose uptake in HepG2 cells by inducing the expression of miR-221. This microRNA binds to PI3K mRNA, thereby inhibiting PI3K/AKT signal transduction. Consequently, PA decreases the expression of glucose transporter type 4 (GLUT4) and inhibits the growth of hepatoma HepG2 cells. In a study by Sai Srinivas et al. (37), it was found that PA inhibits the growth of differentiated human neuroblastoma cells by blocking insulin-induced metabolic activation. This is achieved by inhibiting the activation of the insulin/PI3K/Akt pathway and activating mTOR kinase downstream of Akt. These findings collectively demonstrate that PA exerts its anti-tumor effects by modulating the PI3K/AKT signaling pathway. By inhibiting the expression and activation of key components within this pathway, PA effectively hinders tumor cell proliferation and growth in various cancer types.

### 2.4 The nuclear factor kappa-light-chain-enhancer of activated B cells pathway

NF-κB is a crucial regulator of tumor cell apoptosis, as well as tumor angiogenesis and invasion (38). It has been reported that STAT3 can directly bind to NF-κB and facilitate its nuclear translocation and target gene expression, suggesting that STAT3 is involved in the constitutive activation of NF-κB (39). Interleukin-10 (IL-10) is known to suppress the anti-tumor T cell response and promote tumor growth (40). IL-10 induces the activation of STAT3, and persistent activation of STAT3 is associated with poor prognosis and metastasis (41). Studies have also demonstrated that IL-10 stimulates the epithelial-mesenchymal transition (EMT) process in cancer cells and enhances cancer cell proliferation through the STAT3-NF-κB-IL-10 signaling axis (42, 43). In a study by Fernandes et al. (44), it was found that palmitic acid (PA) inhibited the expression of IL-10, downregulated the expression of STAT3 and NF-κB, and suppressed the proliferation of mouse colorectal cancer (CT-26) cells. These findings suggest that PA can interfere with the STAT3-NF-κB-IL-10 signaling axis, leading to reduced IL-10 expression and inhibition of the downstream pathways involving STAT3 and NF-κB. By targeting this signaling axis, PA exerts anti-tumor effects by inhibiting cancer cell proliferation and potentially suppressing tumor growth and metastasis.

## 3 Induction of tumor cell apoptosis after PA intervention

Tumor cell apoptosis is a crucial process involving the programmed death of tumor cells under both physiological and pathological conditions, playing a significant role in tumor occurrence and development (45). The disruption of the apoptosis mechanism in tumor cells is a key marker of tumorigenesis (46). Consequently, the induction of tumor cell apoptosis serves as a pivotal factor in the selection of anticancer drugs (47). In the field of biomedicine, targeting tumor cell apoptosis holds immense therapeutic potential (20, 48). Small-molecule drugs are commonly employed in clinical treatments as they can trigger tumor cell apoptosis, leading to the elimination of cancer cells.Apoptosis pathways can be categorized into three main types: exogenous apoptosis pathways, endogenous (mitochondrial) apoptosis pathways, and endoplasmic reticulum stress-induced apoptosis (49). By understanding and targeting these apoptosis pathways, researchers and clinicians can develop effective strategies to promote tumor cell apoptosis, thereby improving cancer treatment outcomes.

### 3.1 Tumor suppressor protein p53

The p53 protein is a critical tumor suppressor, and its functional loss is often a prerequisite for the development of cancer (50, 51). In a study by Yu et al. (52), it was discovered that palmitic acid triggers the activation of p53 and the expression of its downstream target genes, namely p21 and Sesn2, in a dose- and time-dependent manner. This activation of p53 was found to promote the apoptosis of colon cancer cells (HCT116). Another study by Saha et al. (53) employed a comprehensive analysis of quantitative proteomics and global phosphorylated proteomics to demonstrate that palmitic acid disrupts the stability of p53 by reducing the expression of ubiquitin-specific protease 7 (USP7). This disruption subsequently facilitates the translocation of apoptosis-inducing factor (AIF) to the nucleus, thereby mediating apoptosis in HepG2 cells.

### 3.2 B cell lymphoma-2 protein family

The high activation of Bcl-2, which exhibits anti-apoptotic effects, has been associated with the occurrence, progression, and prognosis of cancer (54). Consequently, targeting the inactivation of Bcl-2 to enhance apoptosis sensitivity holds great promise for cancer treatment. In a study (55), it was discovered that PA could induce apoptosis of hOSCC cells by upregulating the Bax/Bcl-2 ratio and the expression of Cysteine aspartate-directed proteases-3 (caspase-3). Another study by Cvjeti National et al. (56) demonstrated that PA can trigger Caspase-3-mediated apoptosis in rat insulinoma cells through the activation of p38 mitogen-activated protein kinase (MAPK).These findings highlight the potential of targeting Bcl-2 inactivation and the modulation of caspase-3 activity in inducing apoptosis of cancer cells.

### 3.3 The endoplasmic reticulum

ER plays a vital role in maintaining cellular homeostasis (57). ER stress has been shown to induce apoptosis, a form of cell invasiveness (58). Various markers can be utilized to monitor the state of the ER, with CCAAT/enhancer-binding protein homologous protein (CHOP) being frequently employed alongside pathways such as PKR-like ER kinase (PERK)-Eukaryotic Initiation Factor 2 (eIF2)-activating transcription factor 4 (ATF4) and activating transcription factor 6 (ATF6) (59, 60) PA has been associated with ER stress and apoptosis in pancreatic beta cells (61), hepatocytes (62), and neurons (63) through ER stress mechanisms. Hsiao et al. (21) discovered that PA triggers endoplasmic reticulum stress and cell apoptosis in human neuroblastoma cells by stimulating an increase in phosphorylated eukaryotic translation inhibition factor 2α (a marker of ER stress). Nagata et al. (49) found that PA could induce apoptosis in multiple myeloma (MM) cells by inducing ER stress. Baumann et al. (41) observed in breast cancer cells that PA activates the inositol-requiring enzyme 1 (IRE1)/X-box binding protein 1 (XBP1) and ATF6 axis, leading to a partial ER stress response. However, the PERK-eIF2α axis is not activated, ultimately resulting in CHOP-dependent apoptosis. These studies collectively demonstrate the involvement of ER stress pathways, particularly the activation of CHOP, in PA-induced apoptosis in various cell types. Understanding the intricate relationship between PA, ER stress, and apoptosis contributes to our knowledge of the underlying mechanisms and potential therapeutic applications for diseases associated with ER dysfunction.

### 3.4 Reactive oxygen species

ROS are highly reactive oxygen-containing molecules with a short lifespan, and they play a significant role in inducing DNA damage and affecting the DNA damage response. ROS can trigger tumor cell apoptosis by promoting anti-tumor signaling and initiating oxidative stress (64). Yu et al. (52) demonstrated that PA promotes apoptosis in human colon cancer cells (HCT116) through the induction of reactive oxygen species (ROS) synthesis. This effect is accompanied by a reduction in the levels of important antioxidants such as n-acetylcysteine (NAC) and reduced glutathione (GSH) within the cancer cells. Interestingly, in the context of normal cells, P53 has been shown to play a protective role by safeguarding against DNA damage under low-level cellular stress induced by PA treatment. This protective mechanism potentially contributes to cell survival in normal cells.Another study by Boubaker et al. (65) revealed that PA can enhance lysosome activity and the antioxidant capacity of host macrophages, while inhibiting B16-F10 cells by capturing free radicals and ROS generated from endogenous stress associated with melanin production. Beeharry et al. (66) found that PA can increase oxidative stress by generating ROS, leading to the inhibition of RINm5F cell growth and increased apoptosis. These findings highlight the role of ROS in mediating the effects of PA on tumor cell apoptosis.

## 4 Inhibition of tumor cell metastasis and invasion in response to PA

Tumor invasion and metastasis refer to the process by which tumor cells spread from the primary site to adjacent tissues and eventually to distant parts of the body. This process is pivotal in the development of metastatic cancer and is strongly associated with poor prognosis and disease recurrence. The migratory and invasive capacity of tumor cells plays a critical role in their ability to metastasize (67).

Epithelial-mesenchymal transition (EMT) is a critical process in which epithelial cells undergo a transformation to acquire mesenchymal characteristics (68). In the context of cancer, EMT is closely associated with tumor initiation, invasion, metastasis, and treatment resistance (69). Consequently, blocking the EMT process holds significant promise as an anti-tumor strategy. E-cadherin, a tumor suppressor protein, is involved in regulating EMT and is typically downregulated during cancer metastasis (70). Zhu et al. (29) discovered that PA can modify the expression of various proteins, including protein kinase C zeta (PKCζ), p-integrin β1, and E-cadherin, resulting in the inhibition of metastasis in PC3 and DU145 cells. Moreover, studies have shown that PA can exert an anti-tumor effect by modulating the polarization of tumor-associated macrophages (TAMs) towards an M2 phenotype, thereby impeding EMT in M2 macrophages (44). He et al. (71) demonstrated that PA has the ability to inhibit the proliferation and metastasis of 4T1 breast cancer cells. This effect was achieved by downregulating the mRNA levels of Nfkb1, Snai1, and Stat3, reducing the expression of the EMT marker vimentin, and increasing the expression of E-cadherin. These findings collectively validate the capacity of PA to inhibit EMT and suppress tumor metastasis. The inhibition of EMT holds considerable therapeutic potential for combating cancer progression and metastasis. Further exploration of the mechanisms underlying the modulation of EMT by PA and the development of targeted interventions are warranted to advance our understanding and application of this promising approach in anti-tumor strategies.

## 5 Effect of PA on other factors affecting tumor growth

### 5.1 Metabolic reprogramming

Metabolic reprogramming is a hallmark of cancer, and alterations in energy metabolism are commonly observed in tumor progression. The Warburg effect, characterized by increased aerobic glycolysis, is a well-known metabolic alteration in cancer cells (72) Regarding lipid metabolism and cancer, several studies have investigated the effects of PA and its modulation on cancer cell behavior. Lin et al. (73) demonstrated that PA can impact the aggressiveness of malignant cells in a mouse xenograft model through lipid omics analysis. The precise mechanism underlying this effect may involve changes in cell membrane fluidity and glucose metabolism regulation. Additionally, Sun et al. (74) found that PA can regulate the expression of specific genes involved in fatty acid metabolism, such as stearoyl-CoA desaturase-1 (SCD1), fatty acid synthase (FASN), and elongation of long-chain fatty acids family member 6 (ELOVL6), which are associated with the proliferation of gastric cancer cell lines. The acyl-CoA synthetase long-chain family member 5 (ACSL5) gene, known to play a role in intracellular energy maintenance and carnitine palmitoyl transferase 1A (CPT1A) activity, has been implicated as a key gene in cell proliferation (75). Furthermore, Zhang et al. (76) integrated clinical phenomics, lipidomics, and transcriptomics and found that PA can modulate the expression of the pyruvate dehydrogenase kinase 4 (PDK4) gene and ACSL5 gene in different patterns, leading to the inhibition of proliferation in lung adenocarcinoma or small lung cancer cells by altering cellular energy metabolism. These findings indicate the potential biomedical significance of altered lipid metabolism as a diagnostic marker for cancer cells and suggest the possibility of targeting dysregulated lipid metabolism for cancer treatment. However, it is important to note that further research is needed to fully understand the intricate mechanisms and potential therapeutic strategies related to altered lipid metabolism in cancer.

### 5.2 Enhanced sensitivity to antitumor drugs

Chemotherapy resistance is a significant challenge in cancer treatment, and understanding the mechanisms underlying drug resistance and developing strategies to overcome it are crucial for effective tumor therapy (77). Doxorubicin (DOX) is a commonly used chemotherapy drug for breast cancer; however, its selectivity for tumor cells is limited, and the development of drug resistance is a common issue (78). He et al. (79) conducted a study using poly D, L-lactic acid co-glycolic acid (PLGA) nanoparticles (NPs) encapsulating PA to enhance the efficacy of DOX in breast cancer cells. In mouse models with breast tumors, PLGA-PA NPs exhibited comparable efficacy in reducing primary tumor growth and metastasis when compared to NPs loaded with DOX, PA, and DOX or free DOX. These findings suggest that PA, alone or in combination with DOX, could be a promising strategy for breast cancer treatment. In the case of paclitaxel, another first-line chemotherapy drug, it has been observed that it not only spares macrophages but can also polarize them into classically activated macrophages (M1) (80, 81). Xiang et al. (82) developed palmitic acid-modified human serum albumin (PSA) nanoparticles (NPs) to achieve dual targeting of macrophages and tumor cells for enhanced anti-breast cancer effects.

Short interfering RNA (siRNA) has emerged as a promising tool for targeted therapy in various diseases, including cancer. siRNA molecules, typically around 21-23 nucleotides in length, can specifically bind to complementary mRNA sequences, leading to the degradation of target mRNA and subsequent inhibition of protein synthesis (83–85). In a study by Kubo et al. (86), a PA-coupled siRNA, specifically a 27-nt DsiRNA (C16Dsi27RNA), was developed using a simple synthesis strategy. This C16Dsi27RNA demonstrated enhanced efficacy compared to unmodified Dsi27RNA and cholesterol-conjugated Dsi27RNA in interfering with exogenous enhanced green fluorescent protein (eGFP) and endogenous vascular endothelial growth factor (VEGF) genes in human gastric cancer cell lines (GCIY-eGFP). Moreover, C16-siEGFP, the PA-coupled siRNA targeting eGFP, exhibited significantly higher tumor inhibition compared to unmodified siRNA. Overall, the development of PA-coupled siRNAs provides a promising approach to enhance the efficacy and stability of siRNA-based therapies, opening up new possibilities for targeted treatment options in various diseases, including cancer.

### 5.3 Enhance immunity

The immune system plays a crucial role in defending the body against various diseases, including cancer. Immune cells and their products can have both pro-tumor and anti-tumor effects, and modulating the immune response is an important strategy in cancer therapy (87).Studies have indicated that PA possesses immunomodulatory properties and can enhance the body’s immune response against certain types of cancer (88). Inflammation and cancer progression have been associated with the Toll-like receptor 4 (TLR4) signaling pathway (89). It has been discovered in recent studies that PA can activate TLR4 (90). In line with this, Zhang et al. demonstrated that a combination therapy involving PA and γ-interferon (γ-IFN) inhibits the progression of gastric cancer (GC) by modulating macrophage polarization through the TLR4 pathway (91).

In cervical cancer models, dual PA-coupled toll-like receptor agonists have demonstrated the ability to stimulate M2 macrophage depletion and induce anti-tumor immune responses (92). These findings suggest that PA may have potential as an immune-modulating agent in tumor therapy. It is important to note that the anti-tumor immune effects and mechanisms of PA may vary depending on the specific type of tumor cells involved. Further research is needed to elucidate the specific mechanisms by which PA influences the immune response in different types of cancer.Understanding these mechanisms will contribute to the development of targeted immunotherapies and the optimization of PA-based strategies in cancer treatment.

### 5.4 Autophagy

Autophagy, a process of cellular self-degradation and recycling, plays a significant role in various pathological processes, including malignant transformation, tumor progression, and immune responses in cancer treatment. The modulation of autophagy has been implicated in the development and therapeutic response of cancer (93). Studies have shown that PAcan induce the expression of ER stress marker genes, such as glucose-regulatory protein 78 (GRP78) and CHOP, as well as modulate the expression of autophagy-related genes (microtubule-associated protein 1 light chain 3 (LC3), Autophagy-related gene (Atg) 5, p62, and Beclin) (94). These changes in gene expression promote apoptosis-related gene expression, such as Caspase 3 and BAX, and influence autophagy flux. PA has been found to increase apoptosis and autophagy in Saos-2 cells, suggesting its potential as an antitumor drug by triggering autophagy, which may be particularly relevant in cases where classical apoptosis processes are resistant. Furthermore, PA has been shown to mediate autophagy in hepatoma cells through the regulation of mitochondrial uncoupling protein 2 (UCP2) expression (95). This suggests that PA can modulate autophagy through different molecular mechanisms depending on the specific cellular context. The ability of PA to induce autophagy has significant implications in cancer therapy, as autophagy can impact cell survival, tumor growth, and response to treatment. Understanding the role of PA-induced autophagy and its interactions with other cellular processes will contribute to the development of targeted therapeutic strategies and the optimization of PA-based antitumor approaches. However, it is important to note that the precise mechanisms underlying the autophagy-inducing effects of PA and its therapeutic implications in different cancer types are still being actively investigated. Further research is needed to fully elucidate the complex relationship between PA, autophagy, and cancer pathogenesis, which will guide the development of effective treatment strategies.

### 5.5 Palmitic acid derivatives

Palmitic acid derivatives are compounds derived from palmitic acid through various chemical reactions or modifications. In a study by Liu et al. (96), it was reported that Porcupine (PORCN), in combination with palmitoleoyl-CoA substrate, regulates Wnt signaling and exhibits anticancer properties.Another study by Chen et al. demonstrated that palmitoyl chloride enhances the stability of epigallocatechin-3-gallate (EGCG), and their synthesized compound EGCG palmitate not only induces apoptosis and autophagy in cancer cells but also significantly reduces oxidative stress in normal cells, thereby exerting a protective effect (97). The chemical stability and targeted cytotoxicity of EGCG palmitate make it a promising candidate for anticancer drug development. Although research on the anticancer effects of palmitic acid derivatives is still in its early stages, it has begun to gain attention. It is anticipated that more anticancer drugs based on the properties of palmitic acid will emerge in the near future.

## 6 Conclusion and perspective

**Figure 1:**
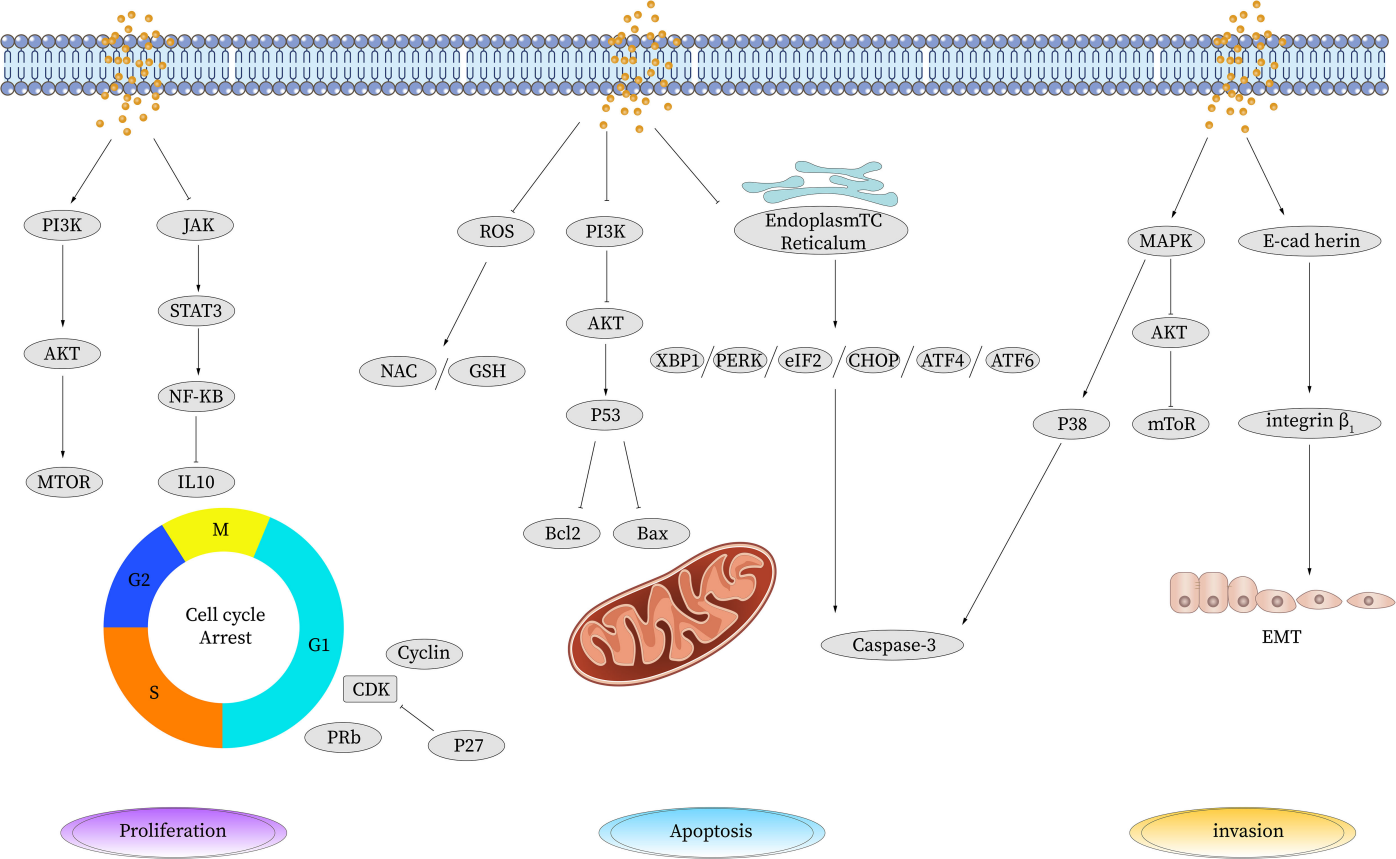
Anti-tumor effect of palmitic acid. PA induces apoptosis by upregulating the expression of P53, reducing the expression of cyclin and cyclin-dependent kinase CDK, inhibiting the expression of Bcl-2, and activating the apoptosis pathway to promote cell apoptosis. PA causes DNA oxidative damage and apoptosis by inducing ROS production. PA can inhibit cell proliferation by increasing the gene expression of TP53 and decreasing the gene expression of CDK, thereby leading to cell cycle arrest. It also reduces the expression levels of IL-10, PERK, and CHOP and the phosphorylation level of JAK/STAT3 and inhibits the JAK/STAT signaling pathway to suppress cell proliferation and metastasis. PA inhibits CDK induced phosphorylation of tyrosinase, thereby inhibiting the activation of NF-κB. PA reduces the phosphorylation of Akt and PI3K and inhibits the activation of the PI3K/AKT pathway. PA inhibits cell invasion and migration by reducing the expression levels of VEGF mRNA and protein and CD34 protein. PA inhibits E-cadherin-induced angiogenesis and inhibits invasion and migration. STAT, signal transduction and transcriptional activator; JAK, Janus kinase; BCL-2, B-cell lymphoma-2; Bax, Bcl-2-associated X; PI3K, phosphatidylinositol 3 kinase; AKT, protein kinase B; mTOR, mammalian target of rapamycin; P53, tumor protein 53; Caspase, cysteinyl aspartate specific proteinase; CDK, cyclin-dependent kinases; ROS, reactive oxygen species; TP53, tumor protein 53; TNF-α, tumor necrosis factor-α; NF-κB, nuclear factor-k-gene binding; IL, interleukin; VEGF, vascular endothelial growth factor; EMT, epithelial-mesenchymal transition; NAC, n-acetylcysteine; GSH, glutathione; eIF, Eukaryotic Initiation Factor; PERK, PKR-like ER kinase; ATF, activating transcription factor; MAPK, mitogen-activated protein kinase.

Indeed, PA has shown great medical potential as a long-chain fatty acid with broad-spectrum anti-tumor effects in various malignancies. It has demonstrated efficacy in inhibiting the proliferation of tumor cells, promoting tumor cell apoptosis, and influencing the cell cycle in gastric cancer, liver cancer, cervical cancer, breast cancer, colorectal cancer, and other types of tumors. The anti-cancer effects of PA primarily involve its impact on the chemical and physical properties of the cell membrane. By increasing the saturation level of membrane lipids, PA influences membrane-related signal transduction pathways, resulting in several beneficial outcomes. These include the inhibition of tumor cell proliferation, induction of tumor cell apoptosis, suppression of tumor cell invasion and migration, and enhancement of the efficacy of chemoradiotherapy drugs. Additionally,PA possesses immune-regulating properties and induces cellular autophagy, which further contributes to its anti-cancer mechanisms. Multiple pathways are involved in the regulation of tumor cell behavior by PA, as illustrated in **Figure 1** and summarized in **Table 1**.

**Table 1:** The antitumor effects of PA.

Mechanism	Cell/tissue type	Dose/concentration	Target	Reference
**Proliferation**	PC3、DU145	5, 10 and 15 μM	pRb、cyclin D1	(29)
	SH-SY5Y	0.3 mM	PKC	(21)
	MGC-803、BGC-823 and SGC-7901	75-150μM	p-STAT3、p-JAK2	(33)
	PC3、DU145	5, 10 and 15 μM	PI3K-P110α and PI3K-P110β	(29)
	HepG2	0.2-0.8mM	miR-221、PI3K/AKT	(36)
	MSN	100、200、500 µM	PI3K/AKT、mTOR	(44)
	CT-26	5、10μM	STAT3-NF-κB-IL-10	(44)
**Apoptosis**	HCT116	116、500μM	p53、p21 and Sesn2	(52)
	HepG2	2.0mM	USP7、P53、AIF	(53)
	hOSCC	10 µg/mL	Bax/Bcl-2	(55)
	LNC	500μM	caspase-3\MAPK	(56)
	SH-SY5Y	0.3 mM	translation inhibition factor 2α	(33)
	MM	0–1000 μM	ER stress	(49)
	SKBR3、BT474	3.2mM	(IRE1)/X-box	(29)
	HCT116	116、500μM	ROS、NAC、GSH	(52)
	B16-F10	25 μM	ROS	(65)
	RINm5F	10 mmol/L	ROS	(66)
**Metastasis**	PC3、DU145	5, 10 and 15 μM	E-cadherin	(29)
	M2 macrophage	5、10μM	STAT3-NF-κB-IL-10	(56)
	4T1	30μM	Vimentin、Nfkb1、Snai and Stat3	(72)
	GCIY-eGFP	50mM	VEGF	(86)
**Metabolic reprogramming**	BGC-823、HGC-27	50、100、200 µM	membrane fluidity and glucose metabolism	(73)
	AGS、MGC-803	5, 10 and 15 μM	SCD1、FASN、ELOVL6	(74)
**Autophagy**	Saos-2	0-800μM	Caspase、BAX	(94)

However, despite its tolerability and safety, most studies on PA have been conducted *in vitro* or using rodent models. Therefore, further research is needed to determine the optimal dosage and safety profile for specific human applications. Additionally, while promising anticancer effects have been observed, the exact mechanisms of action for PA are still not fully understood. The bioavailability of fatty acid components, including PA, is complex and influenced by various factors such as absorption, metabolism, transport, and storage. In addition to its direct effects, the therapeutic mechanism of PA in cancer therapy may be further enhanced through synergistic interactions with extracts, oils, or other substances found in various food sources. Natural products, such as plant extracts and dietary ingredients, are known to contain a diverse array of bioactive compounds, including other fatty acids, polyphenols, flavonoids, terpenoids, and phytochemicals. When combined with PA, these substances have the potential to exert complementary or additive effects on cancer cells, thereby enhancing the overall therapeutic potential of PA. The synergistic interactions between PA and these bioactive compounds can result in improved efficacy and outcomes in cancer treatment.Thus, efficient extraction processes for PA need to be developed to expand its applications in clinical medicine, food, and drug fields.

It is also important to note that PA may exhibit contradictory effects in cancer treatment in certain cases. The effects of PA might vary in different tumor cell lines, emphasizing the need for further research to clarify its mechanisms of action. Furthermore, despite being a natural compound, high doses of PA may have potential side effects and safety risks. Therefore, thorough investigation is necessary to evaluate the risks and benefits of PA in different therapeutic contexts.

In conclusion, while the role and mechanism of PA in cancer therapy require further exploration, it holds promise as a potential therapeutic agent, particularly when combined with other treatment modalities. Future studies should focus on investigating the specific indications, optimal therapeutic dosages, and potential risks of PA. This research will pave the way for the development of safe and effective treatment strategies involving PA in the field of oncology.

## Author contributions

NB conceived and designed the review. XW and CZ wrote the first draft of the manuscript in light of the literature data. Data authentication is not applicable. All authors contributed to the article and approved the submitted version for publication.
